# Focused-Electron-Beam Engineering of 3D Magnetic Nanowires

**DOI:** 10.3390/nano11020402

**Published:** 2021-02-04

**Authors:** César Magén, Javier Pablo-Navarro, José María De Teresa

**Affiliations:** 1 Instituto de Nanociencia y Materiales de Aragón (INMA), Universidad de Zaragoza-CSIC, 50009 Zaragoza, Spain; j.pablo-navarro@hzdr.de (J.P.-N.); deteresa@unizar.es (J.M.D.T.); 2 Laboratorio de Microscopías Avanzadas (LMA), Universidad de Zaragoza, 50018 Zaragoza, Spain; 3 Departamento de Física de la Materia Condensada, Universidad de Zaragoza, 50009 Zaragoza, Spain; 4 Institute of Ion Beam Physics and Materials Research, Helmholtz-Zentrum Dresden-Rossendorf, 01328 Dresden, Germany

**Keywords:** nanomagnetism, focused-electron-beam-induced deposition, nanofabrication, nanolithography, magnetic nanowires, three-dimensional, core-shell, purification, thermal annealing, electron holography

## Abstract

Focused-electron-beam-induced deposition (FEBID) is the ultimate additive nanofabrication technique for the growth of 3D nanostructures. In the field of nanomagnetism and its technological applications, FEBID could be a viable solution to produce future high-density, low-power, fast nanoelectronic devices based on the domain wall conduit in 3D nanomagnets. While FEBID has demonstrated the flexibility to produce 3D nanostructures with almost any shape and geometry, the basic physical properties of these out-of-plane deposits are often seriously degraded from their bulk counterparts due to the presence of contaminants. This work reviews the experimental efforts to understand and control the physical processes involved in 3D FEBID growth of nanomagnets. Co and Fe FEBID straight vertical nanowires have been used as benchmark geometry to tailor their dimensions, microstructure, composition and magnetism by smartly tuning the growth parameters, post-growth purification treatments and heterostructuring.

## 1. Introduction

Three-dimensional (3D) magnetic nanostructures are the playground of a wide range of exciting physical phenomena in nanomagnetism, where curved geometries enable the onset of new types of exotic magnetic configurations, such as topologically protected and chiral magnetic textures [1,2,3,4]. Furthermore, 3D nanomagnets present several key features to postulate a paradigmatic solution to different challenges that the semiconductor industry must confront in the years to come to reconcile continued miniaturization, increasing performance and reduced power consumption [5,6,7]. The transition to 3D nanoarchitectures would overcome the intrinsic areal density limitations of conventional CMOS technologies caused by increasing leakage currents due to quantum effects [8]. They may also reduce the power consumption by storing and processing memory by ultrafast magnetic domain walls or skyrmions driven by low-power spin currents [9,10,11].

Even though the growth of high-purity, narrow 3D ferromagnetic structures may be the gateway to a broad range of opportunities for both fundamental and technological applications, most standard nanolithography techniques lack the flexibility to implement complex 3D structures at the nanoscale. For this purpose, additive manufacturing approaches to nanofabrication present an ideal solution. Among them, focused-electron-beam-induced deposition (FEBID) presents unique properties to become the ultimate 3D nano-printing technique [12]. FEBID is a single-step additive nanolithography technique based on the local decomposition of the molecules of an organometallic precursor gas, adsorbed on the surface of a substrate, thus producing a solid deposit [13,14]. This mechanism is mainly driven by the interaction of the primary beam and the secondary electrons emitted by the substrate with the adsorbed precursor molecules [15]. This basic principle confers a great control on the material deposit, geometry, and growth conditions to design conducting, insulating, superconducting, plasmonic or ferromagnetic structures, with virtually any shape and nanometer resolution, in 2D [16] and 3D [17]. Over the years, numerous types of FEBID deposits have been developed as structural materials [18], for electrical contacting [19], nanosensing [20] or plasmonic structures [21]. In particular, the growth of 3D ferromagnets by FEBID has been fruitful and yielded highly sophisticated architectures. Remarkable applications have been developed in magnetic sensing by functionalization of magnetic probes for magnetic force microscopy [22] and ferromagnetic resonance force microscopy [23] in materials science and biology [24], as magnetically driven mechanical nano-actuators [25], 3D domain wall conduit [26], ferromagnetic designs based on 3D FEBID scaffolds [18], arrays of 3D nanopillars for magnetic logic [27], 3D artificial ferromagnetic lattices [28,29], and magnetically chiral 3D architectures [30]. Further examples of applications of 3D FEBID ferromagnets have been reviewed recently by Fernández-Pacheco et al. [31].

One of the main difficulties of FEBID growth for the design of high-performance nanodevices is to obtain the desired geometry and dimensions with a high level of purity. The presence of a degree of contaminants in the deposit is inherent to the FEBID process, as some of the precursor residues are easily integrated into the deposit together with the active material [32], while secondary electrons emitted away from beam position induce the formation of an extended halo [33]. These drawbacks have motivated a dedicated effort for optimization of FEBID growth conditions, beginning with the choice of the gas precursor. Numerous precursor molecules have been explored for the growth of ferromagnetic materials. A detailed account of the precursors reported in the literature for 2D growth of ferromagnets is beyond the scope of this review, and has been reported elsewhere [34], but it is worth discussing some key aspects. Most of the precursors that have been reported are organometallic complexes based on carbonyl (CO) groups, thus C and O are the expected impurities derived from incomplete precursor decomposition. The most widely used is dicobalt octacarbonyl, Co_2_(CO)_8_, for which 2D deposits with 95 at. % Co content with metallic conduction have been achieved without further post-processing [35], even in halo-free extremely narrow (<30 nm) nanowires [36]. Co(CO)_3_NO has also been tested for 2D FEBID growth, and metal contents of 50–55 at. % have been obtained [37,38]. In the case of iron, the main precursors used are Fe(CO)_5_ [39] and Fe_2_(CO)_9_ [40], and metal contents above 75 at. % have been obtained. Iron precursors evidence the importance of residual gases in the deposition chamber, and 95 at. % purity can be achieved in ultra-high vacuum conditions with Fe(CO)_5_ [41]. The use of heteronuclear precursors allows one to grow alloyed magnetic materials; for instance, the precursor HCo_3_Fe(CO)_12_ has been used to produce Co_3_Fe deposits with metallic contents as high as 80 at. % [42], and has also been grown in 3D [28]. The nickel precursors reported are not carbonyl-based; FEBID deposits based on Ni(C_5_H_4_CH_3_)_2_ [43,44] or Ni(PF_3_)_4_ [43] do not surpass metal contents of 40 at. %. 3D growth of ferromagnets has been attempted with precursors that already provide high purity levels in 2D. In the present work, the precursors used are Co_2_(CO)_8_ for cobalt and Fe_2_(CO)_9_ for iron.

Furthermore, 3D growth implies substantial challenges with respect to 2D growth. As the deposit grows vertically, the geometry of the deposition area changes drastically, and the relevant electron beam interaction is now with the growing deposit, instead of the substrate. Many key parameters for the FEBID process are bound to change with respect to 2D, such as the interaction volume of the electron beam, secondary electron emission, precursor molecules adsorption and diffusion rates, and heat dissipation [45,46]. Moreover, the surface-to-volume ratio also increases significantly, so surface properties become more relevant in 3D nanostructures. This work reviews different possibilities to engineer the geometrical, compositional and magnetic properties of 3D ferromagnetic nanowires grown by FEBID, which can be summarized in three main approaches: (1) fine-tuning of FEBID growth parameters to modify the composition and dimensions [47], (2) the growth of core-shell heterostructures [48], and (3) purification by thermal annealing [49,50].

## 2. Going 3D: Tuning FEBID Growth Parameters

While 3D FEBID growth has achieved a high degree of architectural complexity with the use of computer-aided design models [17], for the optimization of the physical properties of 3D ferromagnetic deposits, the simple benchmark design of a straight vertical nanowire has been used. For this type of geometry, the FEBID growth is performed in spot mode, where a stationary electron beam is focused into a single point of the substrate for a period of time. To facilitate a detailed, local characterization of the physical properties of the 3D deposits, these are grown out on the edge of a commercial TEM Cu grid.

**Figure 1:**
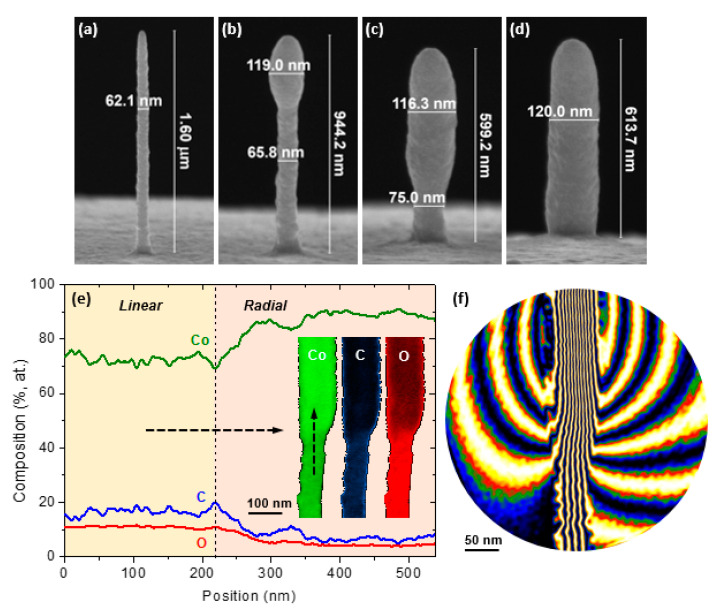
Growth modes of 3D Co focused-electron-beam-induced deposition (FEBID) nanowires. (**a**) Dependence of the transition from linear growth to radial growth with the working pressure increase due to gas injection, ΔP: (**a**) 7.3 × 10^−6^ mbar, (**b**) 6.4 × 10^−6^ mbar, (**c**) 5.9 × 10^−6^ mbar, (**d**) 5.1 × 10^−6^ mbar. (**e**) Compositional dependence with the growth mode, with STEM-EELS elemental maps in the inset, where Co, C and O are depicted in green, blue and red, respectively. The arrows indicate the direction of the elemental line profile. (**f**) Magnetic flux lines around the transition point between sections grown in radial regime (top) and radial regime (bottom). Adapted from Refs. [47,48].

The main growth parameters left are the primary beam energy, the beam current and the precursor gas flux. While the variation of the primary beam energy by itself does not significantly affect the composition, the interplay between the beam current and the precursor gas flux is essential to determine the final properties of the 3D nanowire. Indeed, two growth modes have been evidenced in Co FEBID nanowires, the so-called linear regime and the radial regime [51]. The transition between these two regimes is marked by a sudden change of the nanowire’s diameter, depending on the balance between the beam current and the precursor gas flux. The latter is parameterized through the working pressure, ΔP, defined as the increase of pressure with respect to the base pressure, caused by the precursor gas flux injected during growth. As shown in Figure 1a–d, for a given beam current of 86 pA, a high ΔP of 7.3 × 10^−6^ mbar produces long nanowires with a diameter well below 75 nm, while at low working pressure (ΔP = 5.1 × 10^−6^ mbar), shorter and thicker nanowires are grown, of about 120 nm in diameter. Intermediate values of ΔP give rise to hybrid objects, which evidence linear growth in the early stages up to a certain height, at which the growth transits into radial regime. It is worth noting that for higher beam currents, this transition occurs at higher working pressures, i.e., higher precursor gas fluxes. Thus, the growth rate is determined by the amount of gas molecules delivered (increasing with ΔP), which is known as the precursor-limited regime [52]. Consequently, the nanowires (or segments of nanowire) grown in the linear regime present a high growth rate, expressed in terms of nanowire’s length per unit of time, while in the radial regime, nanowires grow more slowly. The growth mode also reflects on the composition of the nanowire. The radial regime gives rise to the nanowires with the highest Co content, close to 90 at. % Co, while in the linear regime, the values decrease below 70 at. % Co. This tendency is observed even for nanowires that present both growth regimes. There is an illustrative example in Figure 1e, which represents the drastic change in Co content at the transition point between linear and radial regime, determined by electron energy loss spectroscopy (EELS) in scanning transmission electron microscopy (STEM). 

The microscopic origin of this general behavior is evidently complex and requires careful theoretical simulation. However, some aspects can be qualitatively understood as a consequence of the more or less efficient thermal dissipation, and its impact on the gas precursor molecules adsorption/desorption and decomposition. Considering a nanowire growing in the threshold between the linear and radial regimes, at an early stage of growth, the tip of the nanowire is close to the substrate and the heat generated by the electron beam is easily dissipated onto the substrate. However, as the growth continues, the thermal resistance of the deposit increases, and heat is dissipated less efficiently [45]. At this point, the precursor gas may act as a heat-exchange medium. If the working pressure is high enough, thermal dissipation will be sufficient to maintain the linear growth. However, below a certain working pressure, the temperature at the growth point will increase, favoring a faster decomposition of the gas precursor molecules adsorbed and producing a wider deposit. This subtle balance between the heat produced by the electron probe and the capacity to dissipate it is supported by the fact that, at higher beam current (thus, higher temperature at the growth point), a higher precursor flux (thus, more efficient heat exchange) is required to operate in the linear regime. In terms of metallic content, high gas precursor flux favors an incomplete decomposition of the molecules, decreasing the metallic content of the deposit. As a consequence, high working pressure promotes the growth of narrow, though lower purity, 3D Co nanowires.

The beam current is another critical parameter to tailor the composition of 3D nanowires. Co content increases with the beam current, sharply at low currents, up to 80 at. % Co for 200 pA, and moderately at higher currents. As the beam current rises, the precursor molecules are decomposed more efficiently. This also favors the radial regime, as the amount of heat to be dissipated increases, and therefore, the diameters tend to be higher [47].

**Figure 2:**
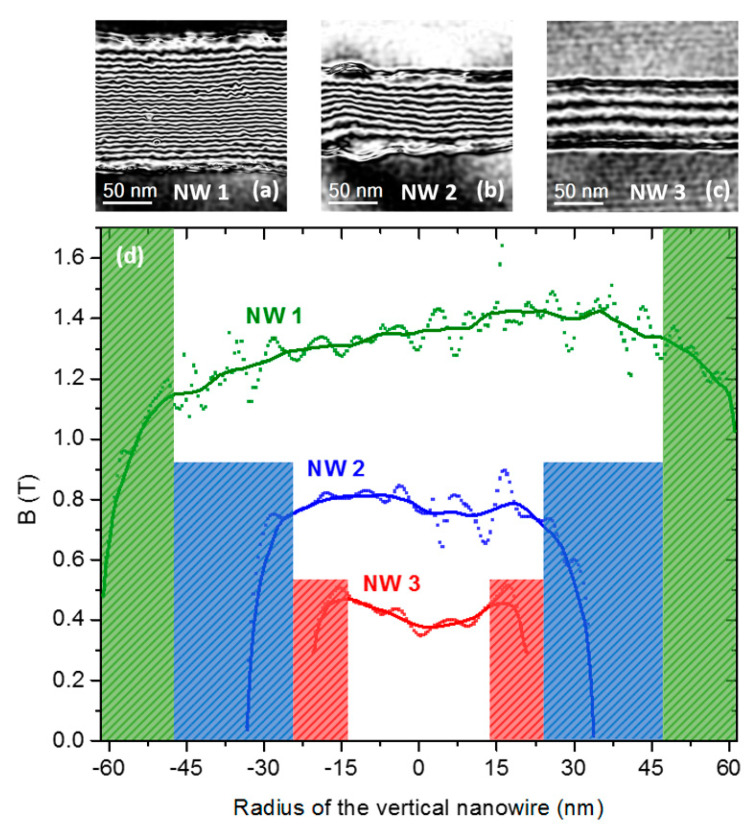
Magnetic properties of as-grown 3D Co FEBID nanowires. (**a**–**c**) Magnetic induction (B) flux maps of nanowires with diameters of 124, 81 and 57 nm, respectively. (**d**) Cross-sectional profiles of B, where the surface regions are marked with vertical color bands. Adapted from Ref. [47].

Of course, Co content of the 3D nanowires have a direct impact on the magnetism. This can be analyzed by off-axis electron holography, which is able to quantify the net magnetic induction of ferromagnetic materials [53]. Figure 1e illustrates qualitatively how the transition from radial to linear regime in a single nanowire causes a reduction of the magnetic flux lines density, which is associated to the lower magnetic induction (B) caused by the reduced Co content. Figure 2 represents the magnetic induction flux produced by 3D Co nanowires with different diameters. The widest nanowire is grown in the radial regime (Figure 2a) with a diameter of 124 nm and a composition of 87 at. % Co, and has an average magnetic induction of 1.33 T, which is 75% of the bulk value (B_bulk_ = 1.76 T). Our estimation does not take into account the fact that the outer surface of the 3D Co FEBID nanowires is oxidized due to air exposure, producing a non-ferromagnetic shell. This oxide layer is highlighted by colored bands in the outer regions where the magnetic induction decays (Figure 2d). The thinnest nanowire is grown in the linear mode (Figure 2c, 57 nm in diameter) and presents a very low magnetic induction of 0.41 T (23% of B_bulk_), in accordance with a much poorer Co content of 41 at. %. This deposit has a much higher surface-to-volume ratio, so the relative contribution of the oxidized surface is remarkable and it is reasonable to think that the inner magnetic induction values are greatly underestimated. Finally, Figure 2b depicts the nanowire with intermediate thickness, grown in the range where radial and linear regimes coexist. This nanowire evidences values of all physical parameters halfway between the two extreme cases: with a diameter of 81 nm, the composition and magnetic induction values are 68 at. %. Co and 0.78 T (44% of B_bulk_), respectively.

**Figure 3:**
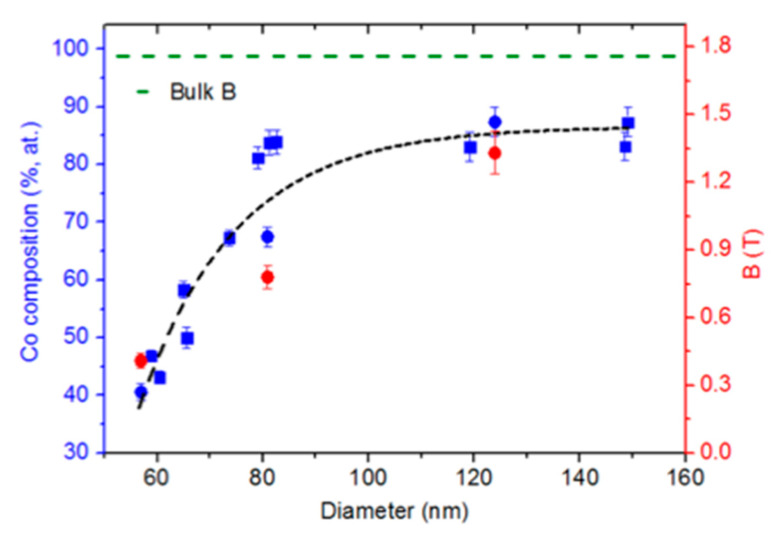
Composition (in blue) and net magnetic induction (in red) of 3D Co nanowires as a function of the diameter. Adapted from Ref. [47].

This study demonstrates that the variation of the main FEBID growth parameters enables the tailoring of the structural, compositional and magnetic properties of the 3D nanowires. They can even be modulated, as evidenced by the observed change in diameter and composition in a single nanowire at the transition from the linear regime segment to the radial regime one. This phenomenon could be exploited to engineer pinning of domain walls of exotic nature [54,55]. However, the key growth parameters and physical properties are mutually dependent, as the Co content, and thus the magnetism, are directly linked to the growth regime, which determines the average diameter and the growth rate. These interrelations are summarized in Figure 3, which represents the dependence of the Co content of nanowires grown in the optimal conditions for a given diameter, together with the net magnetic induction obtained for the three Co nanowires analyzed by electron holography. It is clear that both the composition and magnetic induction inevitably decrease with the reduction of the nanowire’s diameter, and the Co content never surpasses 70 at. % Co for diameters below 80 nm. According to the holography results, this would correspond to a magnetic induction of approximately 0.8 T, which could have a significant impact on the functionality of the nanowires.

Therefore, to produce 3D Co nanowires with a high aspect ratio, diameters well below 100 nm and with good functional properties, namely a high (>90 at. %) Co content and a saturation magnetization close to the bulk value, tuning the basic growth parameters is not enough and post-growth purification procedures are required.

## 3. Purification by Thermal Annealing

The purification of 2D FEBID deposits with the aim of improving their functional properties has been explored for many years. This matter was exhaustively reviewed by Botman et al. in 2009 [32] and many works have been reported later. In situ and ex situ thermal annealing in vacuum [39,56,57], or in a reactive atmosphere [58,59,60,61], substrate heating [62,63] or laser irradiation during growth [64,65], post-growth electron beam irradiation [66,67] or current-induced Joule heating [68], supersonic jet delivery of precursor [69], or the exploration of carbon-free precursors [70] are the most relevant methods used with different degrees of success. However, in the recent expansion of FEBID to 3D [12], few examples of 3D purification can be found in the literature. The most notorious is the post-growth electron-stimulated purification of 3D gold nanodeposits in a water atmosphere to produce virtually pure functional plasmonic nanostructures [21]. In the case of ferromagnetic deposits, the most recent efforts have focused on high-vacuum thermal annealing of 3D Co [49] and Fe [50] nanowires, with different results depending on the precursor and the as-grown metallic content of the deposits.

**Figure 4:**
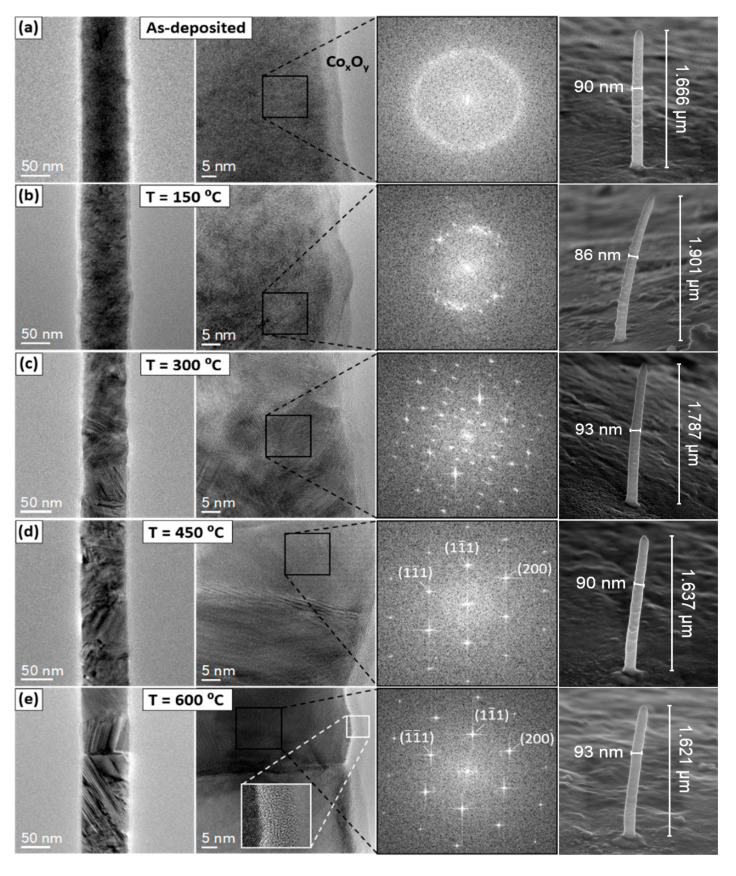
Morphology and microstructure of 3D Co FEBID nanowires as a function of the high-vacuum annealing temperature. TEM, HRTEM images, Fast Fourier Transforms of the squared regions and Scanning Electron Microscopy images of (**a**) an as-grown nanowire and the ones annealed at (**b**) 150 °C, (**c**) 300 °C, (**d**) 450 °C and (**e**) 600 °C. Adapted with permission from Ref. [49]. Copyright 2018 American Chemical Society.

As-grown 3D Co nanowires of approximately 90 nm in diameter, an aspect ratio >15 and a moderate metallic content of approximately 65 at. % Co were used as starting point. These nanowires present a pseudo-amorphous nanocrystalline structure with the presence of a ~5-nm-thick oxide layer (see Figure 4a). Nanowires grown in identical conditions were fabricated in TEM Cu grids, each one of them annealed at 150, 300, 450 and 600 °C for 100 min in vacuum, with base pressures below 4 × 10^−6^ mbar. In terms of the morphology, the annealing process preserves the architectural integrity of the nanowires. As can be seen in the last column of Figure 4, there is no perceptible reduction in volume after the annealing process, which contributes to the preservation of the shape and diameter of the nanowires. This is a key ingredient for the application of these procedures in the design of functional devices, and quite remarkable considering the drastic physicochemical transformations that occurred during the annealing process.

Firstly, the high-vacuum annealing induces the rapid crystallization of the structure. Already at 150 °C, the nanowires evidence the presence of crystals with sizable dimensions, as can be observed in the Fast Fourier Transform of HRTEM images (Figure 4b). Upon further heating, the crystallinity increases (Figure 4c,d) and the average crystal size continues growing, presenting both bcc and hcp structures. Finally, Figure 4e shows how, at 600 °C, only a few Co single crystals that occupy the whole nanowire’s diameter remain, while at the surface, a thin layer of partially graphitized carbon remains as a byproduct of the purification.

**Figure 5:**
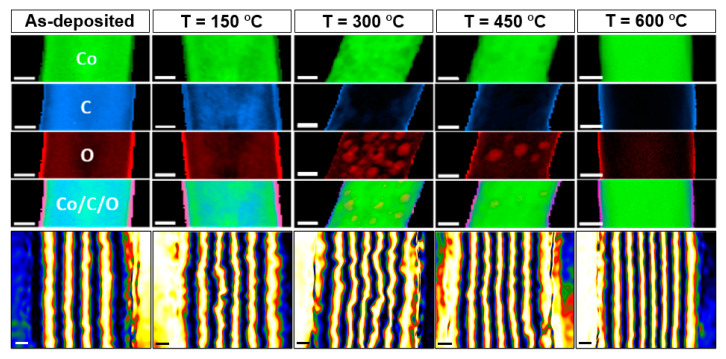
Chemical composition and magnetism of 3D Co FEBID nanowires as a function of the high-vacuum annealing temperature. The first four rows correspond to STEM-EELS elemental maps of Co, C, and O. The last row plots net magnetic induction flux maps obtained in the same nanowires. Scale bars are 20 nm in the STEM-EELS images and 10 nm in the magnetic induction flux maps. Adapted with permission from Ref. [49]. Copyright 2018 American Chemical Society.

The elemental composition of the nanowires follows a peculiar trend, which is illustrated in Figure 5. The as-grown homogeneous distribution of Co, C and O becomes inhomogeneous upon increasing temperature. As the Co content increases, oxygen-rich (most likely, Co_x_O_y_) regions nucleate within the nanowire (at temperatures of 300 °C and 450 °C), while carbon accumulates at the surface. At 600 °C, the inner volume is virtually C- and O-free, with these elements restricted to the surface of the nanowire. This picture agrees with the structural model discussed previously of high-purity, highly crystalline nanowires covered by a thin surface layer of residual contaminants, which gives rise to an overall metal content of approximately 90 at. % Co.

Magnetization evolves similarly to the average chemical composition. The new magnetic induction of the nanowires increases from as-grown B ~ 0.8 T to B ~ 1.35 T for the nanowires annealed at 600 °C, as evidenced from the increasing density of magnetic flux lines in Figure 5. Even though crystallinity has increased, the magnetic configuration remains parallel to the nanowire’s axis in remanence, so the shape anisotropy is still dominant in the purified nanowires.

**Figure 6:**
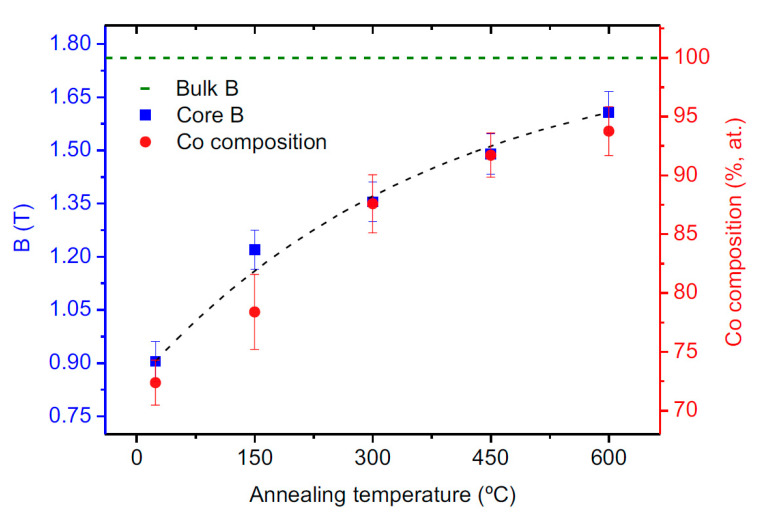
Magnetic induction of the inner magnetic volume and Co composition of 3D Co FEBID nanowires as a function of the annealing temperature. Adapted with permission from Ref. [49]. Copyright 2018 American Chemical Society.

The elemental analysis of the surficial region changes significantly upon annealing (not shown here) [49]. While as-grown and low-temperature annealing present an oxygen-rich surface, a form of Co oxide as a consequence of the exposure to air, upon increasing temperatures, the surface becomes C-rich with a much lower oxygen content. Annealing induces the thermal activation of precursor residues, forming volatile species such as CO and CO_2_ that migrate to the surface and evaporate [57], leaving behind the graphitized carbonous surface, which indeed can be observed in the inset of Figure 4e. This carbonaceous layer actually serves as a protective layer upon further oxidation in subsequent exposure to air. In all cases, all the nanowires are covered by a Co-poor, non-magnetic surface which, depending on the annealing temperature, ranges from 5 to 10 nm. As a consequence, the composition and magnetic induction values of the inner volume of the nanowires are underestimated. After subtracting this contribution and taking into account only the inner magnetic volume of the nanowires, Figure 6 summarizes how the high-vacuum annealing process successfully produces virtually pure crystalline and ferromagnetic Co nanowires with magnetization very close to the bulk value.

**Figure 7:**
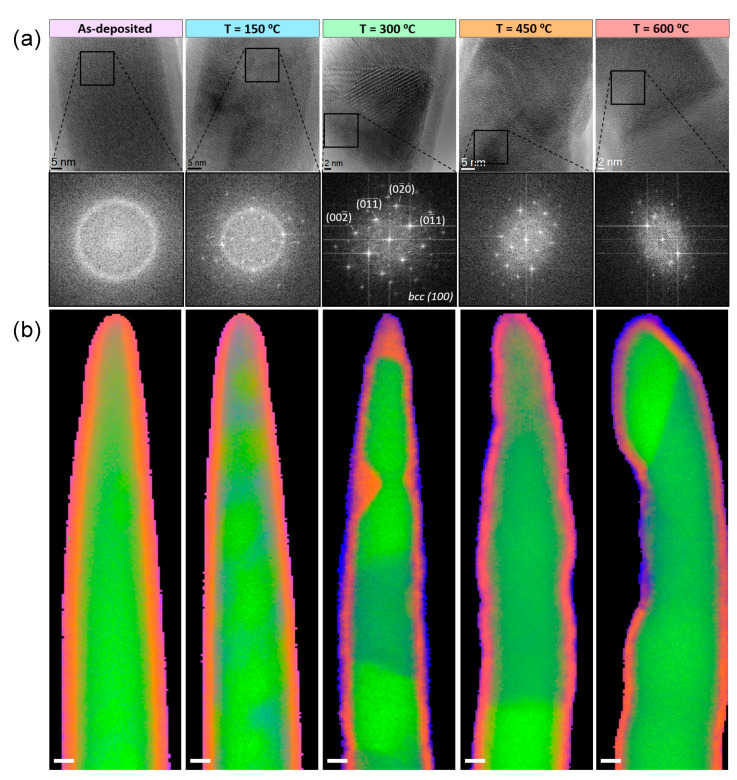
Morphology and microstructure of high-metal-content 3D Fe FEBID nanowires as a function of the high-vacuum annealing temperature. (**a**) HRTEM images and Fast Fourier Transform of the squared regions of the as-grown and annealed objects. (**b**) STEM-EELS elemental maps of the tip of the nanowires, with the spatial distribution of Fe, O and C in green, red and blue, respectively. Undefined scale bars are 10 nm. Reprinted from Ref. [50], Copyright 2019, with permission from Elsevier.

**Figure 8:**
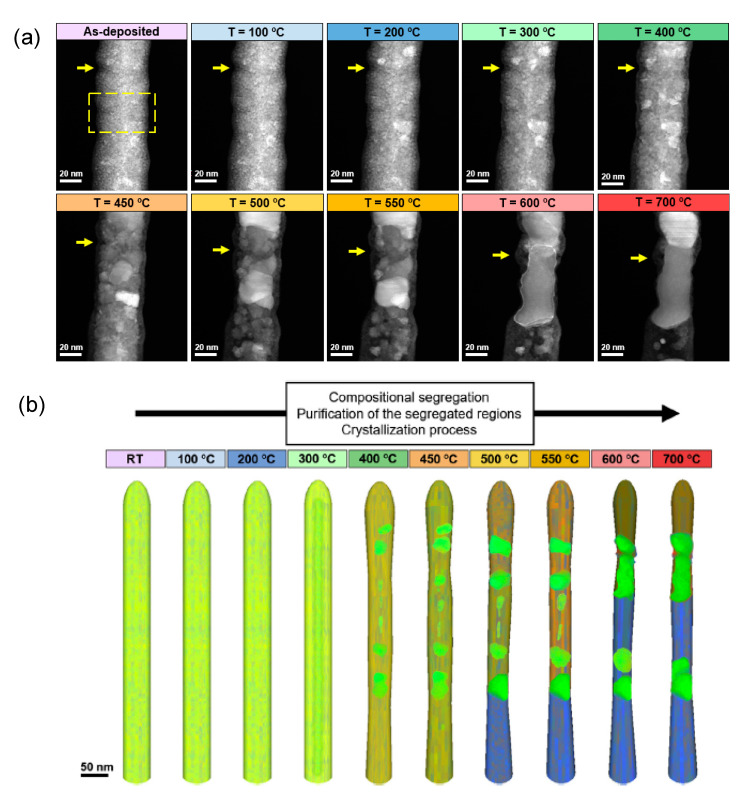
In situ TEM characterization of morphology and microstructure of a low-metal-content 3D Fe FEBID nanowire observed upon high-vacuum thermal annealing. (**a**) STEM images of the same region as a function of temperature. Yellow arrows indicate the same point of the nanowire. (**b**) Sketch of the purification process, where the Fe, O and C spatial distributions are depicted in green, red and blue, respectively. Reprinted from Ref. [50], Copyright 2019, with permission from Elsevier.

This is not an obvious result. A similar annealing procedure has been conducted in Fe FEBID nanowires, as illustrated in Figure 7. For similar growth conditions, 3D Fe nanowires present narrower dimensions (~50 nm in diameter), higher aspect ratio (>50) and a comparable metallic content of ~75 at. %. In this case, a crystallization process similar to the one in FEBID Co occurs at even lower temperatures (wide crystals are already observed at 450 °C), but the purification is not completely homogeneous. While some areas increase their Fe content, it decreases in other regions, giving rise to an heterogeneous object which, in fact, evidences remarkable changes of shape for an already shorter annealing time of 25 min—shorter than in the Co experiments. This indicates that the structural and chemical changes triggered by thermal annealing are not identical for all the materials grown by FEBID, even if the precursors used are similar. This phenomenon has been further investigated by in situ thermal annealing in a transmission electron microscope, in which the whole purification process upon annealing can be monitored in Fe nanowires with similar dimensions, but a lower metallic content of ~40 at. %. Fe. The in situ characterization of the annealing process reveals that, indeed, an inhomogeneous purification takes place. Figure 8 illustrates how high-purity crystalline Fe regions are formed, interspersed between carbonaceous areas. The low purity of the nanowires in comparison with those used for ex situ annealing aggravates this tendency to heterogeneity, presenting remarkable diameter variations which, combined with the extreme compositional variations, change the architecture dramatically and compromise the structural stability of the nanowire.

The different thermal evolution of 3D Co and Fe raises the question of its microscopic origin. Even though the exact mechanisms underlying the FEBID growth and annealing processes are still unclear, the nature of non-metallic residues, for instance, the C:O ratio, is a good indication of the intervening chemical reactions. Barth et al. concluded that a C:O ratio ≥1 might indicate that C-O bonds have been inefficiently cleaved and CO ligands have been incorporated to the deposit, while C:O < 1 suggests the metal oxidation by residual water [71]. In the purification analyses presented in our work, 3D Co corresponds to the first situation, while 3D Fe matches the second one. This is also correlated with the much lower diameter of 3D Fe nanowires; therefore, they are relatively more exposed to ambient atmosphere than Co nanowires. Assuming these considerations, it is very likely that the different chemistry of Co and Fe species in both deposits might be the origin of the different morphological evolution of the nanowires upon thermal annealing.

## 4. Core-Shell Heterostructures

As the diameter of the 3D nanowires is reduced, the nature of the surface becomes significant in terms of the overall physical properties of the object. As discussed in the previous section, due to the synthetic process and the exposure to ambient atmosphere, a surficial region with a thickness in the range 5–10 nm presents a distinct composition, structure, magnetism and, presumably, transport properties with respect to the core. Both as-grown and annealed nanowires present a surface with much lower metallic content due to natural oxidation or accumulation of residual carbon contaminants upon thermal annealing. This is more critical in FEBID Fe, which tends to produce much thinner nanowires—down to 35 nm in diameter [24]—than in FEBID Co. While in a 100 nm-wide 3D Co nanowire, a 5 nm-thick degraded surface represents 19% of the total volume; in a 3D Fe nanowire with a typical diameter of 50 nm, it scales up to 36%. Thus, producing 3D nanowires without a surface degradation might be key to obtain functional objects with lateral resolution below 100 nm with optimum, bulk-like physical properties. As annealing does not fully solve this issue, the growth of a protective cover becomes the sole alternative. While different approaches can be envisaged, the most straightforward solution is the direct growth of another layer of material (i.e., a shell) onto the as-grown nanowire (i.e., the core), prior to air exposure. For this purpose, the growth of a Pt-C FEBID shell is the most convenient, as CH_3_CpPt(CH_3_)_3_ is one of the most standard precursors in SEM-FIB instrumentation and is widely used as a structural or electrical-contact material, with high growth rate and minimal interaction with the deposition area.

**Figure 9:**
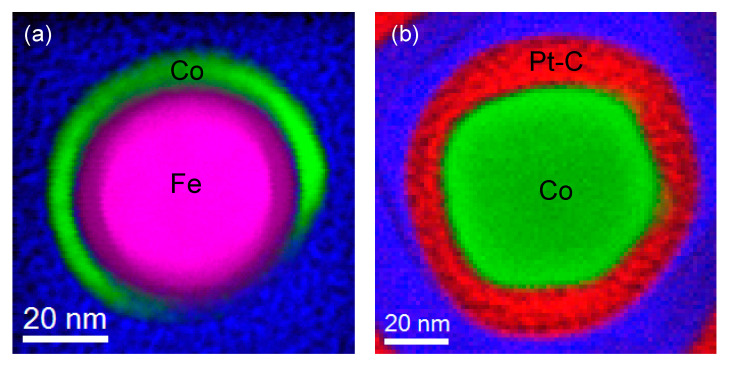
Optimization of 3D core-shell FEBID nanowires. (**a**) Cross-section of a one-step deposition of FEBID Fe@Co. (**b**) Cross-section of a two-step deposition of Co@Pt-C. Adapted from Refs. [48,74].

Therefore, the growth of core-shell Co@Pt-C and Fe@Pt-C by FEBID has been explored. The growth of a homogeneous shell around the magnetic core introduces an additional step to the synthetic procedure. The only successful procedure has been based on the tilt of the nanowire to lay horizontally with respect to the vertical electron beam, thus allowing the deposition of the shell material using a rectangular pattern corresponding to the length of the nanowire. However, a single-step shell deposition is not sufficient. The delivery of gas precursor is highly directional (depends on the actual position of the gas injector system needle), and a higher abundance of precursor molecules should be expected in the face confronting the injector than in the opposite one [72]. Furthermore, the emission of secondary electrons to decompose the precursor molecules adsorbed will be inherently inhomogeneous along the nanowire’s surface, and this compromises the homogeneous coverage of the shell in a single shot [73]. An example of this is shown in Figure 9a, which illustrates the attempt to produce a bimetallic core-shell structure by a single-step shell deposition of Co-FEBID onto a 3D Fe FEBID nanowire. The result is that the back side of the nanowire corresponding to the exit surface of the primary beam remains uncovered. Therefore, a second deposition with the nanowire rotated around its symmetry axis by 180° is required to obtain full coverage, as illustrated in Figure 9b for Co@Pt-C FEBID.

**Figure 10:**
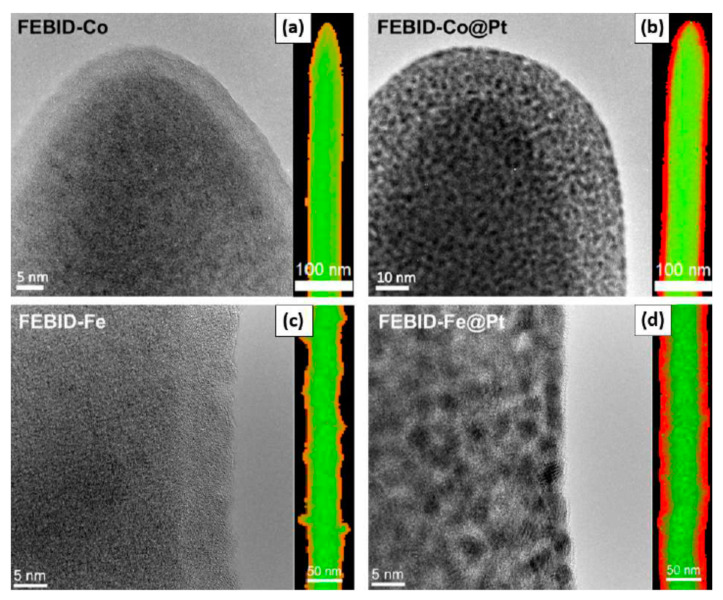
Microstructure and chemical composition of 3D core-shell (**b**) Co@Pt-C and (**d**) Fe@Pt-C FEBID nanowires with respect to the uncoated (**a**) Co and (**c**) Fe nanowires. Each image is accompanied by a STEM-EELS elemental map of the central part of the nanowires where Co(Fe), and O are depicted in green and red, respectively. Adapted from Ref. [48].

Figure 10 qualitatively illustrates the morphology and chemistry of the uncoated 3D Co and Fe cores with respect to their associated Pt-C shell covered counterparts. From the microstructure point of view, the naked magnetic cores, see Figure 10a,c, present the typical nanocrystalline structure covered by a low-density, metal-poor surface. The elemental maps evidence that this is the surficial oxide layer due to air exposure, signaled by the thin metal oxide layer depicted in orange color. In the case of the core-shell structures depicted in Figure 10b,d, the nature of the core is masked by the typical microstructure of FEBID Pt-C, which is an amorphous carbonaceous matrix with small Pt nanoparticles embedded. The distribution of chemical elements of the core-shell structure is radically different from the uncoated ones, as shown by the full green (high metallic content) inner part of the nanowires covered by a vivid red shell, free of Co or Fe. The lack of a transitional region (orangish) between core and shell evidence that there is no Co surface degradation during or after the synthetic process. The oxygen content is also remarkably uniform across the core [48], as these oxygen contaminants are derived from incomplete precursor molecule decomposition and not a result of surface degradation. The quasi-cylindrical geometry of the Co core and the homogeneous coverage of the Pt-C coverage achieved by the two-step shell growth, which was already hinted by the cross-sectional cut shown in Figure 9b, is confirmed all along the nanowire.

**Figure 11:**
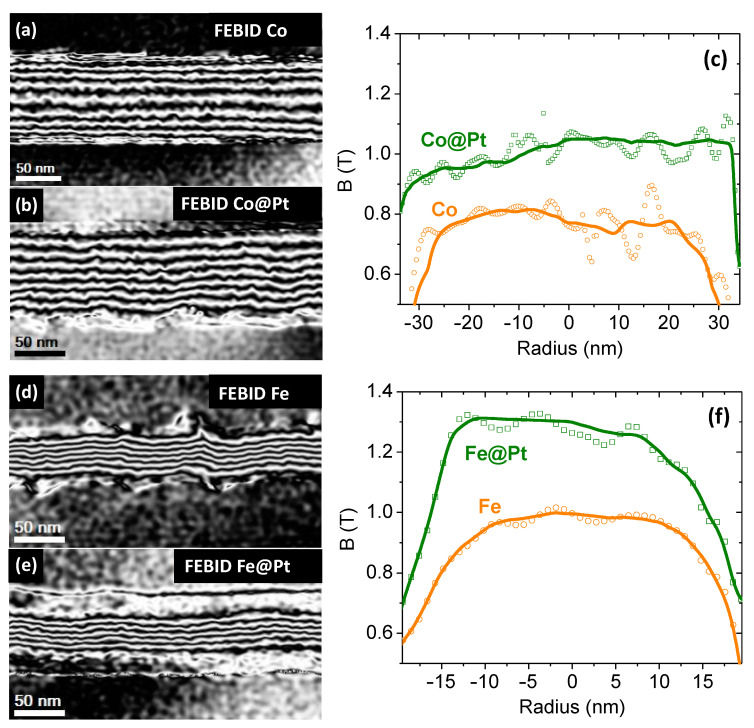
Magnetism of 3D core-shell (**b**) Co@Pt-C and (**e**) Fe@Pt-C FEBID nanowires with respect to the uncoated (**a**) Co and (**d**) Fe nanowires. Magnetic flux maps of each nanowire in the left panel are illustrated by cross-sectional profiles of the net magnetic induction of corresponding (**c**) Co and (**f**) Fe nanowires in the right panel. Adapted from Ref. [48].

The most prominent impact of the core-shell architecture is in the magnetic properties. Again, electron holography is used to perform a quantitative analysis of the net magnetic induction produced by each 3D nanowire [36]. Figure 11 depicts the net magnetic induction flux produced by the set of four 3D nanowire structures and their spatial distribution. This magnetic flux has been quantified, assuming a perfect cylindrical symmetry and normalizing by the nominal diameter of the core. Firstly, holographic images of the uncoated nanowires indicate the suppression or weakening of ferromagnetism in the uncoated nanowires due to air exposure and oxidation—see Figure 11a,d. This is evidenced by the disappearance (in Fe) or decreased density (in Co) of magnetic flux at the surface. Secondly, the magnetic flux of the cores increases with respect to the uncoated nanowires—see Figure 11b,e. This is clearly displayed in the magnetic induction profiles integrated across the nanowires’ diameter, as shown in Figure 11c,f. The increase in magnetic induction is notable, about 20% in the case of Co and 35% in Fe. This values still remain far from bulk values—1.0 T vs. 1.76 T in Co; 1.25 T vs. 2.2 T in Fe. This discrepancy is, however, due to the minute diameter of the cores of the nanowires selected for the magnetic study, which gives rise to particularly low metallic contents in the core, as discussed in Section 2.

## 5. Conclusions and Perspectives

FEBID is an additive nano-manufacturing technique which offers great versatility to fabricate complex motifs and architectural designs at the nanoscale based on numerous materials, and in particular, ferromagnetic materials for high-density, low-power applications as memories, sensors and actuators. The possibilities of 2D FEBID ferromagnetic deposits have been extensively explored, but the expansion to 3D ferromagnetic nanostructures has encountered new challenges in terms of lateral resolution, metallic content and, therefore, the quality of functional properties. Some key advances in the control of growth and optimization of the functional properties of 3D ferromagnetic nanostructures synthesized by FEBID have been reviewed here, using vertical straight nanowires of high aspect ratio as the benchmark.

Understanding the FEBID growth processes of 3D geometries is key to customize their properties. In this regard, one of the main results obtained is the existence of two growth regimes that depend on the subtle balance between the electron beam current and the precursor gas flux, which affects the capacity of the growing nanostructure to dissipate heat during FEBID growth. Depending on these parameters, a radial regime is predominant for low precursor gas flux in which high purity is obtained (>80 at. % Co), but the growth rate is low and the lateral dimensions cannot go below 100 nm in diameter. Higher precursor fluxes enable faster growth, in terms of nanowire length for a given deposition time, and diameters well below 100 nm; however, the metallic content decreases down to 40–50 at. % Co for diameters around 50 nm and magnetization diminishes accordingly. Thus, purification procedures might be required to obtain functional 3D nanostructures with lateral resolutions below 100 nm. High-vacuum thermal annealing has been reviewed as a straightforward approach, as it could be easily implemented right on the deposition chamber. Dissimilar results have been obtained depending on the precursor gas employed and the starting metallic content. 3D Co nanowires based on Co_2_(CO)_8_ precursor achieve virtually pure, crystalline and homogeneous nanowires with bulk-like ferromagnetic properties, with minimum volume reduction, which preserves the original architecture. On the other hand, 3D Fe nanowires grown with Fe_2_(CO)_9_ show a tendency toward phase segregation upon annealing, displaying a mixture of high-purity metal segments interspersed with carbonaceous areas. Both kinds present very different mass density and mechanical strengths, which cause serious shrinkage, drastic diameter variations and, therefore, architectural instability.

The role of surface has been also extensively analysed, and a remarkable degradation of the surface has been observed, mostly due to ambient air exposure. In high surface-to-volume ratio nanostructures such as 3D nanowires, the overall properties depend on surface properties and quality, so a procedure to deposit a Pt-C protective cover by FEBID right after Co or Fe growth has been described. As a result, core-shell nanowires have been designed in which the ferromagnetic cores are free of surface deterioration, with the subsequent improvement in the functional properties, especially in ultrathin nanowires (down to 35 nm in diameter) in which up to 1/2 of the nanowire’s volume is affected by air oxidation.

These findings open new pathways to produce high-quality 3D ferromagnetic nanowires with bulk-like properties and lateral resolution well below 100 nm, and to continue optimizing not only the growth of 3D architectures of nanomagnets, but also of non-magnetic materials. The combination of smart tuning of the growth conditions and thermal annealing in 3D FEBID Co enables the possibility of focusing on the architectural design with the smallest possible lateral dimensions, faster growth and high aspect ratios, at the expense of an optimal composition, which can be improved afterwards by thermal annealing or other methods yet to be explored. The existence of two growth regimes also stimulates the idea of in situ variation growth conditions to produce 3D nanostructures with modulated diameters [75,76]. This idea could be complemented by other strategies that have been recently proposed for diameter reduction and modulation of 3D nanowires, such as the application of local electric fields or the live control of electron beam focus [77]. In cases where post-growth thermal annealing causes severe shrinkage, other means of purification should be explored further to grant architectural stability. For instance, electron stimulation upon reactive atmospheres has been successful in FEBID Au nanostructures [21], and could be explored in Co and Fe, during or after growth.

Once the possibility of fabricating 3D heterostructures by FEBID has been demonstrated, a vast field of research will emerge for optimizing the procedure and inventing novel complex architectures based on the combination of two or more layers or segments of material [54]. In the field of nanomagnetism, the strategy followed for Co@Pt-C nanowires can be reversed, using the same materials to produce ferromagnetic nanotubes on non-magnetic templates, for which attractive magnetic properties for spintronic devices have been predicted in terms of domain wall conduit speed and stability [9] due to the lack of a magnetic core. We have obtained preliminary results indicating that Co FEBID nanotubes are ferromagnetic and nucleate exotic domain walls [74]. The growth of bimetallic nanowires combining Co, Fe and Ni precursors has been hinted in this work, and needs to be explored as a mean to introduce an intrinsic modulation of magnetic properties into the 3D nanomagnet. High-quality interfaces between ferromagnetic materials and high spin-orbit coupling materials such as Pt could be studied for charge-spin conversion in spin-orbitronic applications [78], if proper purification procedures can be developed for 3D FEBID Pt [59]. Considering the available materials, the combination of insulating, conducting, ferromagnetic, superconducting or plasmonic materials is possible to investigate exciting phenomena such as proximity effects with superconductors, designing magneto-optically active nano-objects by combination with plasmonic metals, etc. Finally, the combination of FEBID with other synthetic techniques such as atomic layer deposition could provide infinite possibilities for nano-architectural design of 3D ferromagnetic nanostructures.
